# Examining the cost effectiveness of interventions to promote the physical health of people with mental health problems: a systematic review

**DOI:** 10.1186/1471-2458-13-787

**Published:** 2013-08-29

**Authors:** A-La Park, David McDaid, Prisca Weiser, Carolin Von Gottberg, Thomas Becker, Reinhold Kilian

**Affiliations:** 1 Personal Social Services Research Unit, London School of Economics and Political Science, London, UK; 2 European Observatory on Health Systems and Policies, London School of Economics and Political Science, London, UK; 3 Department of Psychosomatic Medicine, Psychotherapy/Psychiatry, University of Mainz, Mainz, Germany; 4 Department of Psychiatry II, Ulm University, Bezirkskrankenhaus Günzburg, Germany

**Keywords:** Health promotion, Mental health, Economic evaluation, Co-morbidities, Public health

## Abstract

**Background:**

Recently attention has begun to focus not only on assessing the effectiveness of interventions to tackle mental health problems, but also on measures to prevent physical co-morbidity. Individuals with mental health problems are at significantly increased risk of chronic physical health problems, such as cardiovascular disease or diabetes, as well as reduced life expectancy. The excess costs of co-morbid physical and mental health problems are substantial. Potentially, measures to reduce the risk of co-morbid physical health problems may represent excellent value for money.

**Methods:**

To conduct a systematic review to determine what is known about economic evaluations of actions to promote better physical health in individuals identified as having a clinically diagnosed mental disorder, but no physical co-morbidity. Systematic searches of databases were supplemented by hand searches of relevant journals and websites.

**Results:**

Of 1970 studies originally assessed, 11 met our inclusion criteria. In addition, five protocols for other studies were also identified. Studies looked at exercise programmes, nutritional advice, smoking, alcohol and drug cessation, and reducing the risk of blood borne infectious diseases such as HIV/AIDS and hepatitis. All of the lifestyle and smoking cessation studies focused on people with depression and anxiety disorders. Substance abuse and infectious disease prevention studies focused on people with psychoses and bipolar disorder.

**Conclusions:**

There is a very small, albeit growing, literature on the cost effectiveness of interventions to promote the physical health of people with mental health problems. Most studies suggest that value for money actions in specific contexts and settings are available. Given that the success or failure of health promoting interventions can be very context specific, more studies are needed in more settings, focused on different population groups with different mental health problems and reporting intermediate and long term outcomes. There is a need to better distinguish between resource use and costs in a transparent manner, including impacts outside of the health care system. Issues such as programme fidelity, uptake and adherence should also be accounted for in economic analysis. The role of behavioural psychological techniques to influence health behaviours might also be considered.

## Background

Protecting the physical health of people with mental disorders is becoming more prominent in national and regional mental health policies [1-3]. This policy interest comes at a time when a growing number of studies have demonstrated that people with mental disorders are more likely to have costly co-morbid physical health problems than would be seen in the general population [4-7]. Illness, such as psychoses, bipolar disorder and major depression increase the risk of diseases such as obesity, diabetes, cardiovascular disease and chronic obstructive pulmonary disorder [8,9].

Several factors can contribute to poor physical health. Physical illness such as cardiovascular disease, diabetes mellitus and chronic obstructive pulmonary disorder can go under reported and under treated in people with mental health problems [10-12]. Individuals may be reluctant to come into contact with health care services for fear of being labelled as having mental health problems, while clinicians may not place enough emphasis on the management of physical health and provide inadequate assessment, monitoring and care. For instance, one previous review reported that screening rates for metabolic syndrome in people with severe mental health problems remains low [10]. Negative attitudes in some health care professionals may also mean that some physical symptoms are wrongly thought to be a symptom of mental illness rather than an indication of a physical illness [13]. In one recent survey of nearly 800 people living with schizophrenia in 27 European countries, 17% felt that they experienced discrimination when treated for physical health problems [14].

There may also be a lack of incentives in primary care to monitor physical health problems, while the organisation of secondary health care systems in some countries, where mental health services may be largely separated from physical health services, can compound these issues, making the provision of seamless care for both physical and mental health problems more difficult to achieve. The association between poverty and poor mental health may also increase the likelihood that those with more severe mental health problems may live in areas of socio-economic deprivation, which could further impact on their access to and utilisation of health care services

Poor lifestyle behaviours also remain critical contributory factors. This is particularly important given that there may be adverse effects of some medications prescribed to people with mental health problems. Rates of smoking in people with mental health problems are typically much higher than those seen in the general population. In one English population survey, for example, 42% of all cigarettes were smoked by people with mental disorders in 2007 [15]. Reviews covering a range of mental health problems in different population groups in different settings have also highlighted lower levels of physical activity, often combined with poor eating habits and nutritional intake [16-22].

There may also be increased risks of communicable disease such as sexually transmitted infections, including HIV/AIDS, as well as blood borne conditions including hepatitis because of cognitive dysfunction, multiple casual sex partners and unsafe needle sharing among some individuals who also have substance abuse problems [23,24]. The increased risk of homelessness, temporary accommodation or living in institutional settings also compounds the risks of both non communicable and infectious diseases [25,26].

These and other co-morbid physical health problems increase the risk of mortality compared with the general population. This difference in life expectancy can be stark. A study of men and women with severe mental disorders in Denmark, Finland and Sweden reported that they lived 20 and 15 years less respectively than the general population [27]. In an analysis of case registry data in London, and compared with the general population, women with schizophrenia, schizoaffective disorder or bipolar disorder lost between 9.8 and 17.5 years of life, while men lost between 8.0 and 14.6 years [28]. Life expectancy was also lower for individuals in this registry who were being treated for depressive disorders – with a reduced life expectancy of 10.6 and 7.2 years for men and women compared to the general population. Using data from the UK General Practice Research Database on 46,000 people with severe mental illness, Osborn and colleagues also reported a threefold difference in risk of cardiovascular deaths for those between 18 and 49 and an almost twofold increased risk for those aged 50–75 [29].

There is also evidence to suggest that inequalities in all causes of mortality risk appear to have widened between those with severe mental health problems and the general population. One meta-analysis looking at schizophrenia reported a significant increase in standardised mortality ratios using data from twenty-five countries over the period from 1980 to 2006, despite the increased availability of medications that can help individuals better manage mental health problems [30].

Co-morbidity has also been associated with substantially increased economic burden. It compounds the adverse impacts of mental illness with excess costs for the treatment for physical health problems [31-37]. It can also increase the likelihood that individuals will not be able to participate in the labour force, education or look after their families.

All of these factors emphasise the importance of identifying effective approaches to promote and protect the physical health of people with mental health problems, taking into account behavioural, environmental and iatrogenic health risks [38]. Recent reviews indicate an increasing literature on clinical effectiveness studies of interventions to promote physical health and/or treat co-morbid physical health problems in people with mental health problems [39-42].

Potentially measures that are effective in reducing the risk or consequences of co-morbid physical health problems may represent excellent value for money. While there has been some economic analysis of interventions to promote and protect mental health [43], to date comparatively little attention appears to have been paid to the cost effectiveness of physical health promoting interventions for this population. Given that health care budget holders have to make difficult choices on how to allocate scarce resources to mental health and other services, it is crucial to know whether any investment in interventions/programmes to prevent physical health problems in people with mental health problems represents a worthwhile action. What, for instance, are the consequences for resource use in both the health care and related sectors?

### Objectives

Given the importance of this topic the aim of this paper is to conduct a systematic review to identify economic evaluations of interventions intended specifically to promote better physical health and/or prevent physical health problems in people with clinically diagnosed mental disorders. To date only one review appears to have included economic considerations in this area; focused on the effectiveness of psycho-educational, behavioural/exercise/diet modification interventions for people with severe mental disorders it was unable to find any cost-effectiveness studies [41].

## Methods

A detailed review protocol for this analysis is available from the corresponding author on request. This has been developed in line with the established Preferred Reporting Items for Systematic Reviews and Meta Analyses (PRISMA) reporting guidelines [44]. A PRISMA checklist is also provided in Additional file 1.

### Inclusion and exclusion criteria

#### Types of participants

Human participants of any age from studies taking place in high income countries. These studies needed to look at individuals with a primary diagnosis of moderate or severe mental illness, including schizophrenia, bipolar disorder, moderate and major affective disorder and delusional disorder. We only included individuals with alcohol or substance abuse disorders where these were part of a dual diagnosis with one of these mental health problems. We also excluded diagnoses of Alzheimer’s Disease and related disorders, eating disorders and intellectual disabilities, from our analysis.

As our focus here was on disease prevention and health promotion; we excluded studies that looked at the economic benefits of treating physical health problems in people with pre-existing mental and physical co-morbidities. Nor did we look at the economic literature on interventions to prevent mental health problems in people with chronic physical illness such as diabetes or cardiovascular disease. There were no other restrictions on socio-economic or clinical characteristics.

### Types of intervention

Any non pharmacological interventions specifically targeted at promoting the physical health of individuals living with mental health problems. Interventions could for instance include exercise programmes, nutritional advice, smoking, alcohol and drug cessation and infectious diseases prevention. Our analysis was restricted to interventions delivered in countries defined by the World Bank as being high income.

### Types of outcome measure

We included all of the standard economic evaluation methods that have been defined in health economics [45]. In brief, all measure costs in the same way but differ in how they measure economic outcomes. Cost-effectiveness (CEA) and cost-consequences (CCA) analyses report outcomes using natural clinical units e.g. changes in symptom free days; cost-utility analyses (CUA) studies measure outcomes in a common metric either in terms of quality or disability adjusted life years gained; while cost-benefit analyses (CBA) elicit monetary values from the public for different health outcomes. In addition we also included cost-offset analyses (COA), which highlight the potential resource savings of an action without reporting on changes in health outcomes. Studies that looked at economic incentives such as monetary rewards or changes in the price of goods to promote behaviour change were also included within the scope of our review.

### Types of study

All of the economic evaluations described above that bring together cost data with outcome data were eligible for inclusion regardless of whether they were performed prospectively alongside a controlled trial or other quasi-experimental study design or retrospectively using data from a previous effectiveness study. Economic modelling studies that estimated the potential cost effectiveness of interventions by synthesising evidence from trials and other data sources on effectiveness and costs were also eligible. We also recorded study protocol papers reporting current evaluations in order to help identify the current interest in this area, and the way in which the evidence base is likely to develop further.

### Search process

We designed detailed specific systematic search strategies for several health and social science bibliographic databases: PubMed, PsycINFO, CINAHL, Francis, SocIndex and EconLit, covering the period January 1990 to December 2012, with searches run for the period until June 2012 on 7 September 2012, and updated searches for the final six months of the 2012 run on 2 January 2013. Our search strategy for the PubMed database is provided in Additional file 2 as an example of search strategies used. We combined a wide range of phrases for mental disorders with health promotion and public health terms along with terms for economic evaluations and/or specific economic terms /phrases such as cost-benefit. Papers could be in any language, but they needed to have an English language abstract to be potentially eligible for inclusion.

Our electronic search was supplemented by hand-searches of a small number of relevant journals. A limited search of Google Scholar was also undertaken, alongside scrutiny of relevant websites including think-tanks, university economic research groups and some government departments. We also looked at actual examples of programmes and guidelines for the promotion of physical health that were identified separately within our project [39].

References were initially imported into Endnote X5 and duplicates eliminated. Remaining references were initially checked independently by two reviewers, based on their title and abstract to decide if they met our inclusion criteria. In the case of disagreement, further discussions about inclusion/exclusion were made. Full texts of potential matching articles were retrieved and then assessed to determine whether they looked at both the costs and effectiveness of interventions Articles were excluded if full texts did not provide cost or resource data. Data on bibliographic information, the intervention and comparator, duration of study, target population, economic evaluation methods used, empirical study design, cost and resource findings, effectiveness results and synthesis of costs and effects were then extracted from eligible studies into a bespoke Excel data extraction form. We conducted a narrative analysis and review of these studies. We did not plan or conduct statistical meta-analysis or other formal, aggregative synthesis of the results of included studies. All costs were converted to 2010 International Dollars, as well as being reported in their original currency and price year in Table 1.

**Table 1:** Economic evaluations alongside empirical studies for interventions promoting physical health for people with mental health problems

**Author, Year of Publication, Country of Study**	**Intervention (I)**	**Target Population**	**Study Design Type of analysis**	**Summary of main resource and cost results**	**Physical health related effectiveness results**	**Perspective/ Price year**	**Synthesis of costs and effects**
	**Comparator (C)**	**Duration of study**					
Barnett et al. 2008 [55]	I: Stepped smoking cessation programme (computer-assessments of quit readiness; 6 weeks psychological counselling, 10 weeks nicotine replacement therapy; bupropion,extra counselling.	322 cigarette smoking mental health out-patients aged 18+ with a diagnosis of unipolar depression	RCT	The mean costs of intervention were $346. Total mental health care costs in the intervention and control group were $4805 vs $4173. This difference was not significant.	The stepped care group had 5.5.% greater abstinence rate from smoking.(p-value <0.05)	Health care sector	Incremental cost per successful quit $11,496. Incremental cost per life year gained $9,580. Cost effective 74% of time if WTP per successful quit $40,000 .
USA							
	C: brief contact: information on quitting and list of cessation programmes from counsellor.	18 months	CEA			2003 US $	
Chalder et al. 2012 [51]	I: Primary care facilitated physical activity plus usual primary care physician care	361 community dwelling individuals aged 18-69 with first or recent new episode of depression (>= 14 on BDI scale)	RCT	Mean health and social costs per participant in the intervention group were £39 greater, but this was not significant. The mean costs of the intervention for treatment completers were £252. Productivity losses were greater in the intervention group and this difference was almost significant at p=0.05 level	Significantly greater amount of physical activity at 12 months in intervention group Odds Ratio 2.27 p=0.0003.	Health care sector perspective only	Incremental cost per QALY gained of £20,834. 57% probability of being cost effective with WTP threshold of £30,000 per QALY gained. Not considered likely to be cost effective.
England, UK							
					Small but non significant QALY gain of 0.014.		
	C: Usual primary physician care only	12 months	CUA			2009 UK £	
Craig et al. 2008 [58]	I: Integrated management of mental and substance abuse disorders by specially trained case managers in community mental health teams (CMHTs).	127 community dwelling mentally ill patients with comorbid substance use disorders treated by 40 specially trained and supervised case managers in CMHTs and105 community dwelling patients receiving usual case management from 39 case managers in CMHTs.	RCT	Total mean costs in the intervention and control groups were £18,672 and £17,639. This difference was not significant.	No impact on substance use levels between the groups at 18 month follow up, but small positive impact on mental health status	Health care and criminal justice sectors	No synthesis was reported as there was no significant difference in costs or substance abuse levels between the two groups.
UK							
	C: Standard case management in CMHTs	18 months	CCA			2004 UK £	
Gusi et al. 2008 [49]	I: Primary care initiated supervised walks with a group in a park or forest tracks for 50 minutes, 3 times per week plus simple diet advice.	127 overweight, moderately obese or moderately depressed community dwelling older women.	RCT	The incremental cost of the exercise programme plus best care, relative to best care was €2250.	Body Mass Index (p<0.003)	Health care sector	Incremental cost per QALY gained: €311. 99.9% probability of being cost effective if WTP of just €600 per QALY gained.
Spain					Exercise: 29.7->29.4		
					Control: 30.6-> 30.8.		
					The mean incremental Quality Adjusted Life Years gained was 0.132 (95% CI:0.104-0.286)		
	C: The standard	6 months	CUA			2005 €	
	“best primary care” : routine care in general practice and a recommendation of exercise						
Johnson-Masotti et al. 2000 [48]	Two interventions:	Community dwelling people with severe mental illness being treated on an outpatient basis at risk of HIV.	Modelling	The total costs of intervention include staff compensation, materials, transportation, overhead, and participants’ opportunity costs. Average cost per person:	Infection averted per 100 clients	Societal	Advocacy training group (A) was most cost effective for men with incremental cost per QALY gained of $48,585. For women single session intervention is cost saving
	I: A multi-session small group intervention (M)						
					Men		
USA	I: Advocacy training (multi-session that taught participants to act as safer sex advocates to their peers).(A)			Single session: $178	S: 0.041		
				Multi-session: $629	M: 0.087		
				Advocacy training: $786	A: 0.138		
					Women		
					S:0.098		
					M: -0.041		
					A: 0.019		
					QALY gains not documented		

	C: A single session, one-on-one HIV/AIDS education intervention (S)	3 months	CUA			1998 US $	
Morse et al. 2006 [59]	Two interventions	149 homeless people treated on an outpatient basis having a wide range of severe mental illness with substance disorder (i.e. dual disorder diagnosis).	RCT	The mean total costs for the IACT ($48,764) and control group ($41,726) were significantly less than those for the ACTO group ($71,211) (p<0.05).	There were no differences between treatment groups in substance use.	Health care	Costs and outcomes were separately reported.
	I: Integrated Assertive Community Treatment (IACT)						
USA							
	I: Assertive Community Treatment Only (ACTO)						
	C: Standard care	24 months	CCA			2001 US $	
Murphy et al 2012 (early online) [50]	I: 16 week tailored programme of exercise delivered in a leisure centre supervised by a qualified exercise professional. Plus subsequent 8 month telephone contact by exercise professional.	2,160 community dwelling sedentary individuals having coronary heart disease (CHD) risk, and/or mental health problems (mild anxiety, depression/ stress disorders)	RCT	Incremental cost for the mental health or mental health plus CHD group was £596 but this was not significant. There was a small significant improvement of 0.0058 QALYs gained in this group.	CHD group reported significantly higher levels of physical activity, but no difference for those referred wholly or partially for mental health reasons. The mental health group did have statistically significant improvement in depression/anxiety.	Public sector	Incremental cost per QALY gained for whole population £12,111. 89% probability of being cost effective at £30,000 per QALY gained. £10,276 per QALY gained for mental health or mental health and CHD group.
Wales, UK							
	C:Usual care plus information on benefits of exercise and location of local facilities	12 months	CUA			2009 UK £	
Pinkerton et al. 2001 [47]	I: Small group HIV prevention programme in community mental health clinics, focusing on sexual communication, condom use skills, and motivation to practice safer sex.	87 community dwelling women at least being 18 years old with a psychiatric diagnosis of mental illness.	Modelling	Intervention cost per participant: $679. Saved $13,830 in HIV-related medical care costs. The cost per 100 women was $67,910, a net cost of $54,080 costs avoided in medical care costs. For sexually active women only, there were $22,284 in avoided medical care costs per 100 women.	For full sample, intervention averted 0.064 infections and saved 0.40 QALYs. For sexually active women only, 0.104 infections were averted and 0.64 QALYs saved.	Societal	For full sample, cost per QALY saved: $136,295.
USA							
							For sexually active women only, $71,367 per QALY saved.
	C: Standard health promotion programme without inclusion of HIV	6 months	CUA			1999 US$	
Rosenberg et al. 2004 [60]	I: Specialist brief programme delivered in community mental health centres to reduce risk of blood borne infectious disease.	173 community dwelling people with serious mental illness	A ‘before and after’ pilot study at one urban and one rural community mental health centre	Intervention costs per person ranged between $194 and $262.	Increased motivation to reduce risk behaviour such as HIV and hepatitis (p<0.01). But no actual decrease in self-reported risk behaviour.	Health care	Concluded that pilot study supports feasibility and efficacy of intervention.
USA							
	C: No controls – change in knowledge and risk behaviours post intervention	6 months	CCA			2002 US $	
Rosenberg et al. 2010 [61]	I: Specialist brief programme delivered in community mental health centres to reduce risk of blood borne infectious disease	236 community dwelling people with severe mental illness and co-occurring substance use disorder largely from ethnic minority groups.	RCT	Intervention cost per person: $541 including $234 for blood tests.	People in the intervention group were more likely to be tested for HBV and HCV , and immunised against hepatitis A and hepatitis B, to reduce their substance abuse. However, they showed no decrease in risk behaviour.	Health care	Costs and outcomes were separately reported.
USA							
	C: Enhanced treatment as usual.	12 months	CCA			US $	
Timko et al. 2006 [46]	I: Community residential facility acute support programme for dual disorder people	57 community dwelling and 173 hospital with dual psychiatric disorder and substance abuse diagnosis	RCT	Mean health care costs for the community group were $21,966 compared with $33,188 in the hospital group. This difference was not significant.	The community group had significantly improved Addiction Severity Index Scores compared to the hospital group. 26% of the community group were in remission compared with 16% of the hospital group. This was not significant	Health care	Costs and outcomes reported separately, but noted that mean costs for patients in remission in community group of $12,174 were less than half those of hospital group.
USA							
	C: Hospital inpatient acute support programme for dual disorder people	12 months	CCA	For those patients successfully in remission from substance abuse cost		2003 US$	

## Results

**Figure 1:**
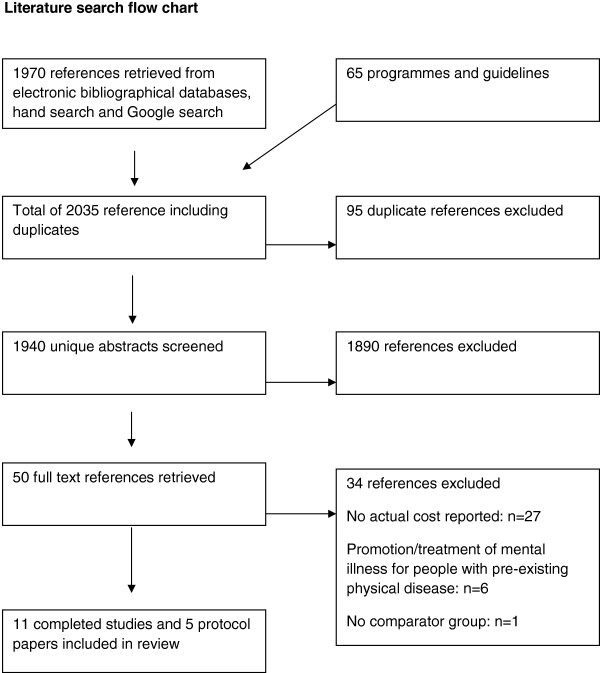
Literature search flow chart.

As Figure 1 indicates, our search process resulted in 1970 references being identified. 50 articles were subsequently retrieved based on the relevance of their abstracts. Of these, six were excluded as they focused on interventions to improve co-morbid mental disorders for people with pre-existing physical disease, which was outside the scope of our current review. 27 studies which initially appeared to be relevant were eventually excluded as no actual detailed costing and resource use was reported and another was excluded because of a lack of information on any comparator group. This left 11 completed studies which are summarised in Table 1. Table 2 summarises information on a further five protocol papers for ongoing or planned economic evaluations that were identified and which should meet our inclusion criteria when completed.

**Table 2:** Protocol papers for current economic evaluations

**Bibliographic information**	**Intervention (I)**	**Target Population /Duration**	**Study Design Type of analysis**	**Cost results**	**Physical health related Effectiveness results**	**Perspective /Price year**	**Synthesis of costs and effects**
	**Comparator (C)**						
Bonevski et al. 2011 [57]	I: Client centred, caseworker-delivered cessation support intervention for a socially disadvantaged population.	200 community dwelling socially disadvantaged smokers including people with mental health problems attending a community social service centre	RCT	Resources and costs of interventions and impacts on health service use	Changes in smoking behaviour	Health system only	Not stated
Australia							
	C: Information on smoking cessation and telephone number for Quitline	12 months	CCA				
Carter et al. 2012 [52]	I: 12 week preferred intensity aerobic exercise, with motivational coaching and support	Community dwelling people aged 14-17 already in contact with health care services with depression	RCT plus focus group analysis	Boundaries not stated but will use Client Service Receipt Inventory to record health care service use.	Changes in depression using Children’s Depression Inventory	Not stated	Incremental cost per QALY gained
England, UK							
					QALYs using EQ-5D		
	C: Usual care	9 months	CUA, CEA		Physical Activity Intensity (Borg Scale)		
Kruisdijk et al. 2012 [54]	I: 6 months exercise therapy or Nordic walking plus usual care	People aged 18-65 with diagnosis of depression or bipolar disorder who are being treated on an inpatient or outpatient basis	RCT	Resource use and costs determined using Trimbos/iMTA Questionnaire for Costs associated with Psychiatric Illness (TIC-P)	Risk factors for metabolic syndrome. Fitness and Physical Activity. QALYs using EQ-5D	Health care use and productivity losses	Incremental cost per QALY gained
The Netherlands							
	C: Usual care	12 months	CUA				
Stockings et al. 2011 [56]	I: Multi-modal smoking cessation intervention (brief motivational interviewing plus range of post discharge support for up to 16 weeks.	200 smokers aged 18+ in an inpatient facility with acute mental health problems	RCT	Resource use and costs needed to deliver intervention	Changes in smoking behaviour. Use of alcohol and other substance abuse. Mental wellbeing	Health system only	Not stated
Australia							
	C: Hospital smoking care only includes a referral to Quitline on discharge.	6 months post discharge	CCA				
Verhaeghe et al. 2012 [53]	I: 10 week health promotion intervention (psycho-educational and behavioural group sessions, supervised exercise, individual counselling) targeting physical activity and diet plus usual care	People aged 18 – 75 with mental disorders living in sheltered housing. 201 in intervention; 83 in control group 9 months (RCT)	Cluster RCT and Markov Modelling to extrapolate risk of CVD and diabetes over 10 years	Intervention costs plus costs of health care utilisation	BMI, Waist Circumference, body weight, fat mass, QALYs using SF-36	Health system	Incremental cost per QALY gained
Belgium							
	C:Usual care	10 years (model)	CUA				

Of completed studies seven were from the USA, with three from the UK and one from Spain. Seven studies focused on psychosis and/or bipolar disorder. Three of these studies focused on interventions to tackle substance misuse and its consequences in people with dual diagnosis, while four reported on infectious disease reduction strategies. The remaining four studies focused predominantly on people with depression and/or anxiety disorders, looking at lifestyle modification interventions in terms of a smoking cessation programme, as well as three programmes focused on the promotion of physical activity.

Looking at the type of economic evaluation technique used, five studies were cost-consequences analyses, with five cost-utility analyses and one cost-effectiveness analysis. No cost benefit analyses were identified. Eight of these economic evaluations were performed prospectively alongside randomised controlled trials. One of two retrospective studies used a non-randomised quasi-experimental study design to look at the adoption of the rehabilitative treatment facilities for military veterans [46]. Two studies explored the costs and benefits of treating co-morbid HIV infections by making use of modelling techniques [47,48]. We now look at these studies in more detail.

### Lifestyle-based interventions to promote physical health

One key concern focuses on promoting a sustained increase in physical activity and improved nutrition among people with mental health needs. We identified three studies looking at different physical activity interventions. One Spanish study evaluated a walking-based, supervised exercise programme for low-income older women with moderate depression and/or obesity [49]. 55 women were randomly allocated to a 50 minute supervised walking programme, three times per week for six months, in addition to dietary advice plus best primary care alone, compared with 51 women receiving usual primary care and a recommendation to exercise. Outcomes examined included changes in mean Body Mass Index (BMI) and changes in quality of life. After six months mean BMI decreased from 29.7 to 29.4 in the walking group, but increased from 30.6 to 30.8 in the usual care group (p<0.003). Supervised walking was highly cost effective at $449 (€311, 2005 prices) per QALY gained, with a 99.9% probability of being cost effective assuming a cost per QALY threshold of just $865 (€600 2003 prices). These QALY gains may be conservative if the increased level of physical activity can be maintained over the longer term.

In Wales the National Exercise Referral Scheme has also been subject to economic evaluation [50]. Sedentary individuals aged 17 or older with coronary heart disease risk factors and/or mild anxiety, depression or stress were randomised to receive a 16 week tailored exercise programme delivered by exercise professionals using motivational interview techniques to encourage the long term sustainability of behaviour change. During this time and for the subsequent eight months they were followed up by phone by the exercise professional to help reduce the risk of relapse. The intervention was found to significantly increase physical activity in those with coronary risk only; it did not have an impact on physical activity in those with mental health problems, although there were significant reductions in their depression or anxiety status, suggesting that they may have benefited from social contact arising from participation in the scheme. The cost per QALY gained for the mental health group was $15,808 (£10,276, 2009 prices) compared with $18,631 (£12,111, 2009 prices) for the whole mental health and coronary heart disease risk group. There was an 89% of being cost effective for the whole study population with a cost per QALY threshold of $46,150 (£30,000, 2009 prices).

A smaller scale randomised controlled trial looking at professionally facilitated physical activity for people with depression compared to usual primary care was undertaken in England [51]. While the central aim was to look at the impact of exercise on depression, for which no impact was found, the study reported that the rate of sustained physical activity in the intervention group was more than double that of the control group at 12 month follow up. This finding is important given the challenges in motivating people with depression to engage in physical activity. Therefore while the intervention was not considered to be cost effective as an intervention to tackle depression, with little impact on recorded QALY outcomes during the lifetime of the study, the intervention may have potential in the longer term to be cost effective, if the increase in physical activity in this population group can be sustained and further benefits to physical health realised.

The number of lifestyle behaviour modification evaluations will grow as we also identified three protocol papers. In England a published protocol describes an economic evaluation alongside a trial of self-determined intensity one hour per week for 12 weeks aerobic exercise programme for community dwelling people aged 14–17 with depression [52]. Two of the protocols will consider people either in residential or inpatient settings. One is for a cost utility analysis alongside a cluster randomised controlled trial comparing the use of a ten week health promotion programme versus usual care for people with a range of mental health problems living in sheltered housing in Flanders, Belgium [53]. The programme, which is being delivered to more than 200 people, includes group-based educational sessions, supervised exercise and individual counselling. While empirical data will only cover a six month follow up period, modelling will be used to estimate the impacts of sustained behaviour change on risks of cardiovascular disease and diabetes over a ten year period.

The second study in the Netherlands is for a controlled trial on the use of regular hour=long running therapy or Nordic walking programmes over six months by people of working age with depression or bipolar disorder being treated on an inpatient or outpatient basis [54]. Impacts on metabolic risk factors, physical activity rates and mental health will be assessed over one year. All three of these studies will measure several outcomes including incremental costs per QALY gained.

### Programmes to reduce smoking, alcohol and substance abuse

Another key concern is to tackle addictive behaviours such as smoking, alcohol and illicit substance abuse in people with mental health problems. However we were only able to identify one cost effectiveness study looking specifically at smoking cessation interventions for people with mental health problems. This study compared a stepped cessation programme (three computer-mediated assessments of readiness to quit smoking, six psychological counselling sessions, up to ten weeks nicotine replacement therapy, and offer of sustained-release bupropion and two additional counselling sessions) in outpatient care settings with brief care for smokers with depression [55]. After 18 months the stepped care group had a 5.5% greater chance of ceasing smoking (P<0.05) at a cost per quit of $13,519 ($11,496, 2003 prices) or $11,266 ($9,580, 2003 prices) per life year gained. In sensitivity analysis, applying a cost effectiveness threshold of $40,000 per successful quit, there was a 74% chance that the smoking cessation programme would be cost effective.

We also identified two study protocols from Australia related to smoking cessation interventions. One is looking at the integration of multi-modal smoking cessation strategies at an Australian inpatient facility and post-discharge, including an analysis of cost impact [56]. The second will evaluate the effectiveness and cost consequences of smoking cessation advice provided to socially vulnerable smokers, including those with mental health problems, at a community social services centre [57].

We also identified studies looking at the economic case for tackling the harmful effects on health of substance use in individuals with a dual mental disorder / substance abuse diagnosis. In south London, one evaluation looked at the costs and effectiveness of an integrated care programme using case managers trained to deal with substance use disorders in people with severe mental illness [58]. After 18 months, no significant differences in costs, alcohol consumption or cannabis use were observed between the integrated care group and waiting list controls. A lack of continuity in case management due to high staff turnover, diverting resources to staff retraining and retention mechanisms may have hampered the intervention, while the duration of the study was also felt by the authors not to be long enough to achieve significant changes in substance use.

In the USA, a small study with 149 participants also explored costs and outcomes of integrated treatment for both mental health needs and substance abuse by the health care team plus Assertive Community Treatment (IACT), compared with Assertive Community Treatment Only (ACTO) or standard care (SC) for homeless people with a dual diagnosis [59]. While the study reported no significant difference in substance abuse or mental health outcomes, the IACT and ACTO groups were significantly more likely to be in stable housing (P=0.03); they were also significantly more likely to be satisfied with their care (P=0.03). Over 24 months, IACT and SC had significantly lower costs than ACT (p value not reported). The ACTO group’s costs may have been higher because they did not follow their own protocol and made much use of external outpatient mental health services rather than using their own team psychiatrist.

Another randomised controlled trial from the US evaluated costs and outcomes for 230 people with a dual diagnosis of co-morbid substance use and psychiatric disorders initially assigned to either a hospital or community residential facility (CRFs) acute care programme [46]. Over one year, patients in the CRF group had Addition Severity Index (ASI) scores that were significantly lower for both alcohol use (p<0.01) and drug use (P<0.05) compared to the hospital group. Patients with moderate psychiatric disorder severity showed better ASI scores for alcohol use in CRFs (p<0.05), however patients with the highest levels of severity had better outcomes in the hospital group, although this difference was not statistically significant. While there was no statistically significant difference in overall health care costs between the groups, for those patients successfully in remission, health care costs in the CRF group were $14,317, ($12,174, 2003 prices) which was just over half the costs of similar patients in the hospital care group. While acknowledging that these results should be interpreted with caution due to small sample size, the authors suggested that shifting more patients with less severe disorders away from hospital care to community-based care could potentially lead to cost-savings, especially if in addition to a reduction in substance abuse other beneficial aspects of community care in terms of its less restrictive nature and promotion of social inclusion, were also considered.

### Programmes to reduce the risk of infectious disease

People with dual diagnosis in particular are more likely to be exposed to infectious disease such as HIV/AIDS, hepatitis B and hepatitis C. Four economic studies of interventions to reduce the risk of infectious disease were also identified. Some were targeted at high risk populations such as people with both severe psychiatric disorders and substance abuse.

The costs and outcomes of the brief STIRR (Screen, Test, Immunise, Reduce risk, and Refer) intervention delivered by a mobile specialist team to prevent or detect and treat blood-borne diseases in people with serious mental health problems have been evaluated. Initially an uncontrolled pilot study in two community mental health centres in New Hampshire [60] reported significant improvements in the levels of knowledge about blood-borne infectious diseases (p<0.01) and in motivation for prevention before and after the intervention (p<0.01), but no decrease was found in self-reported risk behaviours. The cost of the programme ranged from $233 to $315 ($194 to $262, 2002 prices) per participant. Subsequently the costs and outcomes of the programme were assessed in a randomised controlled trial delivered to a dual diagnosis urban population [61]. Compared with enhanced treatment as usual, there were modest significant positive changes in the DALI (Dartmouth Assessment of Lifestyle Instrument) scores for alcohol use (p<0.022). Moreover, the STIRR group showed significant improvements in clinician-rated Drug Use Scale scores (p<0.034). However, there were no significant changes in HIV knowledge or in risky behaviours. Programme costs per participant were $551 ($541, 2008 prices) per participant (2008 US$). However, the costs of care as usual, as well as any consequent impacts related to health care resource utilisation were not reported.

In the US, Johnson-Masotti et al. performed a cost-utility analysis of three cognitive-behavioural HIV/AIDS risk reduction interventions for people with severe mental illness, synthesising data using mathematical modelling for a three-month period [48]. The interventions were a single session (one-on-one) HIV education intervention, a multiple-session small group intervention, and a multi-session group intervention for teaching peer advocacy skills. Gender differences were observed in interventions that were most cost effective, with advocacy training for men having an incremental cost per QALY gained compared to a single session intervention of $62,875 ($48,585, 1998 prices) a figure that would be considered cost effective in a US context. For women, while the single session intervention was actually cost saving compared to doing nothing, the multi-session intervention was less effective at higher cost, while investing in advocacy training would not have been cost-effective at an incremental cost of $603,062 ($465,994, 1998 prices) per QALY gained.

Another US study looked at the cost-effectiveness of a nine session small-group intervention to prevent HIV-infection in women with mental disorders [47]. It suggested that a targeted approach may be more cost effective than a population wide strategy. For the whole study population a cost per QALY gained of $173,828 ($136,295, 1999 prices) was seen, compared with a cost per QALY of $91,020 ($71,367, 1999 prices) for high risk women, who had been sexually active in the previous three months. Although still well above the often suggested threshold value of $50,000 per QALY gained in the USA [62], there may have been potential for an even more targeted strategy to be cost effective.

## Discussion

There is a growing body of clinical effectiveness studies on programmes to prevent somatic diseases in people with mental disorders [39-42,63,64]. We have also indicated that the importance of protecting physical health is to be found in different mental health policy plans. While there is an evidence base on the cost effectiveness of many health promoting interventions for the general population, our review indicates that there is still very little information available on the cost-effectiveness of these interventions for people with pre-existing mental health problems.

One previous review found no cost-effectiveness studies of lifestyle interventions on the physical activity and eating habits of people with severe mental disorders [41], while a recent Cochrane review was unavailable to find a single randomised controlled trial reporting evidence on the effectiveness or cost effectiveness of guidelines advising on the monitoring of physical health in people with severe mental health problems [65]. Similarly a review of programmes to promote the physical health of people with mental health problems was only able to find some brief information on the commercial costs of obtaining manualised programmes rather than any evidence of their cost effectiveness [39].

Smoking cessation has also been highlighted as a key goal for the protection of physical health in people with mental health problems, given the higher rates of smoking in these populations compared with the general population [66]. While there is an extensive literature on the cost effectiveness of different smoking cessation strategies for the general public, indicating that highly cost effective smoking cessation interventions are available [67,68], we were only able to identify one economic evaluation of a smoking cessation study targeted at people with mental health needs [55].

We were also only able to identify three economic evaluations of interventions to encourage physical activity, all of which focused on depression and or anxiety disorders rather than also being targeted at people with other severe mental disorders. There is a need to have more studies focusing also on addressing physical health promotion in individuals with severe mental illness, particularly given their higher risks of metabolic syndrome as a result of medication use.

This situation may however be changing; two physical activity related studies in our review were published in 2012 and there are three study protocols looking at the cost effectiveness of healthy lifestyle promoting interventions, two of which will target people with more severe mental illness who are being treated in inpatient and residential settings. A further two study protocols we identified will look at smoking cessation interventions, including one for residents of an acute psychiatric inpatient care facility.

Overall, most of the studies we identified suggest that there is an economic case for protecting the physical health of people with mental health needs, and most report incremental cost effectiveness ratios such as cost per smoking quit achieved or cost per QALY gained.

### Strengthening the literature

There remain substantial limitations and research gaps with the existing literature. Here we briefly discuss issues around uptake and behaviour change; population sub-group analysis; the timeframe of studies; study perspective; sample size; transferability to different contexts; better understanding of issues of fidelity in implementation; and the potential for economic modelling.

### Uptake and behaviour change

Firstly, there was little exploration in this analysis of factors that either inhibit or promote sustained behaviour change. This is of particular importance when considering health promoting interventions which require regular behaviour change and/or participation in an activity over a period of time to have an impact, e.g. exercise and diet programmes. Economic analyses need to consider the extent to which these uptake issues apply to interventions to address physical co-morbidity in this population and what additional costs are incurred as a result of effective measures taken to promote better uptake [69]. It may well be the case that interventions that currently do not appear cost effective may be seen in a more favourable light if resources were invested in better tailoring of programmes and interventions to people with mental health needs.

This also raises the possibility of looking at the cost effectiveness of techniques from behavioural psychology or economics to influence individual behaviour patterns and perhaps ‘nudge’ people towards different types of behaviours. Recent reviews of this literature in respect of people with mental health problems are tentatively positive [70,71], requiring further research not only on effectiveness but also on the costs of implementation.

In this context nurses and other health care professionals such as physical health and lifestyle trainers that routinely work closely with people with mental health problems can focus more on looking at factors that may encourage greater sustained participation in health promoting activities [72]. Strengthening the training of professionals in somatic care, as well as going beyond the absence of mental disorder to consider the benefits of positive wellbeing, can only be helpful for lifestyle monitoring and behavioural change [73]. Better training will also reduce the likelihood that these professionals ‘avoid’ dealing with physical health issues through a lack of expertise.

### Sub-group analyses

It is also important to consider cost effectiveness for population sub-groups as cost effectiveness ratios for the population as a whole may not reflect the cost effectiveness of the intervention for specific population groups, for instance related to age, gender, diagnosis or living conditions [74]. Few of the studies undertaken to date have looked at sub-groups, although there can be very different conclusions drawn on what is cost effective, as in the case of the evaluation of HIV risk reduction programmes for men or women [48]. It may also be the case, for example, that it might be easier to encourage individuals to take up physical activities when they are living in residential accommodation which includes a gym or other sporting equipment, compared with individuals who are living independently in the community and may not have easy access to this equipment. In the same way it may be more challenging for individuals to eat healthily if they are living in areas with little easy access to fresh fruit and vegetables, but many fast food restaurants [75]. This is also important when considering the implications for health inequalities of any intervention. Health promoting interventions for the general population can in some circumstances widen health inequalities within the population because of different rates of uptake and capacity to benefit [76].

### Study timeframes

We also know comparatively little about the long term effectiveness and therefore long term cost effectiveness of interventions. Most studies had a short-term follow-up period between three and 12 months. Only three studies covered longer periods from 18 to 24 months. We have noted that health promotion interventions may require regular long term use to ensure sustained behaviour change, let alone have a sustained impact on physical health. In a shorter time frame, studies may have to rely on intermediate outcomes such as abstinence from smoking or harmful drinking or changes in dietary behaviour. These intermediate indicators may not always immediately translate into anticipated health gains, although there may be short term benefits to be flagged up e.g. from social capital gained through socialisation and networking with other participants in an exercise class. Economic analysis should also consider the economic benefits of any improvements in mental health arising from health promoting interventions.

### Study perspective

It is also important to consider the impact of study perspective on the cost effectiveness. Some of the costs of health promoting interventions might be borne outside the health care system, e.g. local government may be responsible for some aspects of sports and recreation activities, while there will also be external economic benefits such a potential reduction in the need for informal care support from family members and a reduction in time spent out of work for people of working age. Some of these broader impacts may make the case for investment stronger. Better physical health may remove one barrier to maintaining or gaining a job, improving self-care and self-management skills, as well as improving quality of life and sense of social inclusion in the local community. However most studies we identified focused solely on the perspective of the health care budget holder alone and did not consider these broader impacts. Only four studies looked at costs from a broader viewpoint, including costs to the criminal justice system [58], the public purse [51] and the opportunity costs incurred by study participants [47,48].

### Sample size

Another challenge is the lack of statistically significant differences in costs between interventions and comparators in a number of studies. This is by no means unique to this literature, but it means that studies may have been powered to detect significant differences in effect size between interventions and control groups, but not any significant differences in cost [46,48,49,59,61]. In studies with small sample sizes, skewed costs driven by a few unusually costly individuals in one community can also distort potential average cost per participant [59,77].

### Transferability across contexts

Most studies are concentrated in the US; but regardless of where a study is set how generalisable are findings to other contexts? Uptake and use of interventions may be influenced by the organisation, structure and culture of health care systems in different countries. Even within a country, especially where there are significant differences in structures between regions, e.g. between American states, cost effectiveness findings on programmes may not be easily generalisable [59]. In the case of the Spanish walking for health programme, the high rate of recruitment (79%) had an influence on programme cost effectiveness. A low cost intervention, a letter from a general practitioner sent to women in the target population, was sufficient to encourage participation in the programme [49]. In countries where primary care does not play such a pivotal role, different, potentially more expensive, methods of engaging with the target population may be required.

It is also important to explicitly report resources used separately from their costs in order to aid future adaptation of study results [69][78] and to be aware of the fidelity in the way that programmes have been implemented in different contexts; a lack of fidelity in complying with recommended practice protocols meant that Integrated Assertive Community Treatment was reported to be less costly than standard Assertive Community Treatment in one study in our analysis [59].

### Fidelity in implementation

Process and context evaluation alongside economic analyses is also crucial to monitor the fidelity in implementation of interventions. Consultation with experts can be used to get a sense of the feasibility of delivering an intervention to a minority population group, for example. In the STIRR programme, cultural competence experts/ethnographers, who were specialised in urban, African-American culture checked any potential cultural issues with pilot participants from ethnic minority backgrounds before delivering the intervention to the larger study population [61]. Discussions with local experts can also help to identify any potential differences and resource requirements that are likely to be observed when adapting an intervention to a different context.

### Using models

Economic models can be used to synthesise data on costs and benefits; crucially they can be used to project long term costs and benefits well beyond the duration of any empirical study. Yet they appear to have been rarely used for interventions for this client group thus far, although one of the study protocols identified in this review will model ten year impacts on cardiovascular disease and diabetes of a health promotion intervention [53].

Cost and resource parameters and potential health promotion pathways and outcomes can also be adapted in models to take account of different infrastructure contexts, cultural factors or different levels of engagement in different population groups. Different scenarios can be constructed to take account of different probabilities of uptake and participation in population sub-groups, for instance accounting for differences in the severity of symptoms for different mental disorders or accommodation status. Impacts on quality of life and other outcomes can also be varied in modelling analyses. The potential long term benefits to health of sustained change in health promoting behaviours can also be projected in models.

Sensitivity analysis with these models can be used to explore the minimum level of effectiveness that would be required for an intervention to be cost effective in different country contexts. This in turn can help decision makers determine whether piloting a health promoting approach developed elsewhere is feasible. Such analyses might also help in better targeting health promoting interventions, or in adapting them to the needs of different population sub-groups.

### Limitations of the review

Although our review was not restricted to English language materials, no studies in other languages were identified. One potential bias may be that we did not search any non-English language bibliographic databases, nor did we search for terms in languages other than English. We also excluded studies such as exercise programmes for people with mental health problems which at first sight intuitively appear to be relevant. These were excluded because they did not report physical health outcomes, however, much may be learnt on the acceptability of exercise programmes from these studies. In addition, quite a few economic evaluation studies excluded were for interventions to prevent and treat infectious diseases such as HIV/AIDS, hepatitis B and hepatitis C for people with substance abuse without any official diagnosis of mental illness. Given the high level of co-morbidity between mental illness and substance abuse, it may be the case that much can be learnt from these studies.

We also did not look at the cost effectiveness of any pharmacological developments that may reduce any risk of adverse events such as weight gain and metabolic syndrome, from antipsychotics or antidepressants. Although excluded from our analysis, we did identify one such study [79]. This is an area that also merits further future analyses, including exploration of combined health promotion/pharmacological interventions. It may also be the case that useful data from specialist early intervention teams for mental health may have been overlooked, given that these teams often include physical health specialists. To date however, economic studies of these teams have focused on impacts on mental health alone [80].

The small number of studies identified, coupled with limited diagnostic differentiation, also means that we have to be very cautious in our interpretation of the findings of this review. Interventions looking at physical activity and smoking cessation have been targeted at people with depression and anxiety disorders; the merits of these interventions in promoting the physical health of people with other severe mental illness need to be assessed. Equally, actions to prevent substance abuse and infectious disease have concentrated on severe mental illness and need to be explored with other population groups.

Despite these limitations we believe we have conducted the most comprehensive review to date of economic evaluations for physical health promotion in people with mental health problems. Economic evaluations covering interventions to promote physical activity and better dietary behaviour, discourage smoking, substance and alcohol consumption and reduce the risk of blood borne disease specifically in populations with mental health needs, both within inpatient facilities and in the community, have been identified.

## Conclusions

There is a very small, albeit growing, literature on the cost effectiveness of interventions to promote the physical health of people with mental health problems. Most of these studies suggest that value for money actions in specific contexts and settings are available. What is clear however is that as the success or failure of health promoting interventions can be very context specific, more studies are needed in more settings, reporting outcomes in a common metric, such as quality of life years gained, and showing resource use and costs in a transparent manner, including costs beyond the health care system.

In the short-term, economic modelling techniques might be better utilised to explore possible costs and benefits under different scenarios and over different time horizons, making use of a wide range of sensitivity analyses to account for uncertainty in data parameters. Such models can synthesise data from existing effectiveness reviews [40,41]. In the mid to longer term it would be helpful to at least collect data on resource use and cost impacts alongside effectiveness data in new trials. This can facilitate better comparability across different interventions and ultimately help policymakers in the public health arena to prioritise resource allocations to specific preventive strategies.

Finally it should never be forgotten that economic evaluations are not conducted in isolation. Apart from cost-effectiveness, it is crucial to look at barriers and facilitators to intervention by being mindful of the findings of any process evaluation, so as to take into account issues such as programme fidelity, uptake and adherence. The role of behavioural psychological techniques to influence health behaviours and mitigate any adverse impact on health inequalities might also be considered. Looking at the success or failure of the organisation of any system to promote physical health can also be the subject of economic analysis, for instance in terms of different types of multidisciplinary team work, links between primary and specialist care services, and in the co-ordination and continuity of mental and physical health care services.

## Abbreviations

ACTO: Assertive community treatment only; AIDS: Acquired immune deficiency syndrome; ASI: Addition severity index; BMI: Body mass index; CBA: Cost benefit analysis; CCA: Cost consequences analysis; CEA: Cost effectiveness analysis; CHD: Coronary heart disease; COA: Cost offset analysis; CRFs: Community residential facilities; CUA: Cost utility analysis; DALI: Dartmouth assessment of life instrument; DALY: Disability adjusted life years; EQ-5D: EuroQOL; HIV: Human immunodeficiency virus; IACT: Integrated assertive community treatment; QALY: Quality adjusted life years; RCT: Randomised Controlled Trial; SF-36: Short-form (36) health survey; STIRR: Screen, test, immune, reduce risk, and refer; UK: United Kingdom; US: United States; WTP: Willingness to pay.

## Competing interests

DM is an associate editor of BMC Public Health. The authors declare that they have no other conflicts of interests.

## Authors’ contributions

ALP, DM, TB and RK conceived the manuscript. DM and ALP were mainly responsible for search strategy design, study selection and analysis and drafting the manuscript. PW, CVG, TB and RK separately searched for evidence on the effectiveness of interventions. ALP and DM wrote the first draft of the manuscript. PW, CVG, TB and RK contributed to the writing of the manuscript. All authors agree with the manuscript results and conclusions. All authors read and approved the final manuscript.

## Pre-publication history

The pre-publication history for this paper can be accessed here:

http://www.biomedcentral.com/1471-2458/13/787/prepub

## Supplementary Material

Additional file 1 PRISMA Checklist. Click here for file

Additional file 2 PubMed/Medline search strategy. Click here for file
