# Supplementary material for: Examining the cost effectiveness of interventions to promote the physical health of people with mental health problems: a systematic review
Source: BMC Public Health. 2013 Aug 29;13:787. doi: 10.1186/1471-2458-13-787 (PMC3765875; doi:10.1186/1471-2458-13-787)
Supplement: Additional file 2 — PubMed/Medline search strategy. [file 1471-2458-13-787-S2.docx]

**Additional file 2**

**PubMed/Medline Search Strategy**

1. Mental Disorders/
2. Mental Disorder$.ti,ab
3. Mental Illness.ti,ab
4. Psychiatric Disorder$.ti,ab
5. Depressi*.ti,ab
6. Anxiety.ti,ab
7. Schizophreni*.ti,ab
8. Bipolar Disorder.ti,ab
9. Psychotic Disorder.ti,ab
10. Psychosis.ti,ab
11. 1-10/OR
12. Costs and Cost Analysis/
13. Cost-Effect*.ti,ab
14. Cost-Benefit.ti,ab
15. Cost-Utility.ti,ab
16. Cost-Consequence.ti.ab
17. 12-13/OR
18. Health Promotion /
19. Health Education /
20. 15-16/OR
21. Public Health / Economics.sh
22. 11 AND 18
23. 11 AND 14 AND 17
24. 19 OR 20
25. Animals/
26. Humans/ AND Animals/
27. 25 NOT 26
28. 24 NOT 27
29. Abstract Available
30. Publication Date 01/01/1990-31/12/2012
